# RNA interference in the Asian Longhorned Beetle:Identification of Key RNAi Genes and Reference Genes for RT-qPCR

**DOI:** 10.1038/s41598-017-08813-1

**Published:** 2017-08-21

**Authors:** Thais B. Rodrigues, Ramesh Kumar Dhandapani, Jian J. Duan, Subba Reddy Palli

**Affiliations:** 1 Department of Entomology, University of Kentucky, College of Agriculture, Food and Environment, Lexington, KY USA; 2 USDA ARS Beneficial Insects Introduction Research Unit, Newark Delaware, USA

## Abstract

Asian Longhorned Beetle (ALB) *Anoplophora glabripennis* is a serious invasive forest pest in several countries including the United States, Canada, and Europe. RNA interference (RNAi) technology is being developed as a novel method for pest management. Here, we identified the ALB core RNAi genes including those coding for Dicer, Argonaute, and double-stranded RNA-binding proteins (dsRBP) as well as for proteins involved in dsRNA transport and the systemic RNAi. We also compared expression of six potential reference genes that could be used to normalize gene expression and selected *gapdh* and *rpl32* as the most reliable genes among different tissues and stages of ALB. Injection of double-stranded RNA (dsRNA) targeting gene coding for inhibitor of apoptosis (IAP) into larvae and adults resulted in a significant knockdown of this gene and caused the death of 90% of the larvae and 100% of adults. No mortality of both larvae and adults injected with dsRNA targeting gene coding for green fluorescence protein (GFP, as a negative control) was observed. These data suggest that functional RNAi machinery exists in ALB and a potential RNAi-based method could be developed for controlling this insect.

## Introduction

The Asian longhorn beetle (ALB), *Anoplophora glabripennis* (Coleoptera Cerambycidae), is a polyphagous wood boring insect native to China and Korea and has invaded the United States, Canada, and several countries in Europe^1^. This beetle has been documented in more than 100 different species of trees and recognized as a globally important invasive species^2^. For example, the potential economic impact for 2016 in the United States would have reached $889 billion if the discovered populations have not been controlled by the current eradication program^3^. Early detection, quarantine, and eradication efforts are currently still the main strategy against ALB. However, the costs and environmental concerns from the removal or chemical treatment of large numbers of host trees in or near the newly infested area call for the development of novel approaches to effectively control this invasive pest. The recent completion of *A. glabripennis* genome and transcriptome sequencing has provided valuable tools to develop novel molecular approaches for controlling ALB^3–5^.

RNA interference (RNAi) is a gene silencing mechanism triggered by double-stranded RNA (dsRNA)^6^. RNAi is being developed as a novel pest management method by targeting and silencing essential genes involved in insect survival^7, 8^. The RNAi machinery in siRNA (small interfering RNA), miRNA (microRNA) and piRNA (PIWI-associated RNA) pathways has been identified in insects^9, 10^. These pathways differ in many aspects, such as the RNA precursor molecules and specific enzymes involved in their processing. While piRNAs are produced independent of Dicer (RNase III enzymes) activity, miRNAs require Drosha (RNase III-family enzyme) to process the stem-loop dsRNA structures of its precursor molecules. Dicer 1 and 2 enzymes are also required to process long RNAs to miRNAs or siRNAs respectively. The processed small RNAs are loaded on to the silencing complexes with the help of dsRNA binding-proteins (dsRBP). In insects, these dsRBPs are specific to each Dicer; Loquacious (Loqs) for Dicer 1, R2D2 for Dicer 2 and Pasha for Drosha^11^. Argonautes (Ago) are main proteins of the silencing complexes composed of two domains, PAZ domain involved in dsRNA binding and PIWI domain responsible for RNase activity. In all three pathways, specific Ago mediates both target recognition and silencing of miRNA (Ago-1), siRNA (Ago-2), and piRNA (Aubergine, Aub and Ago-3). The presence or absence of the core RNAi genes does not seem to be the only reason for the variable efficiency of RNAi observed among different insects. Several other factors such as the digestion of dsRNA by dsRNase enzymes present in the gut lumen and hemolymph, the dsRNA transport into and within the cells and its systemic spread and even the expression levels of RNAi genes have been shown to influence RNAi efficiency among insect tested^12–17^.

To determine the knockdown efficiency of dsRNA, reverse transcription quantitative PCR (RT-qPCR) is often used. Although RT-qPCR is a robust method with a combination of high specificity, accuracy, and sensitive detection, this method is influenced by several factors, such as the stability of reference genes, quantity and purity of RNA used, and primer efficiency^18, 19^. A critical step that may compensate most of the variability at both RNA and PCR level is the selection of a reference gene constitutively expressed across the samples and treatments under study^20^. Recent studies have suggested that there is no universal reference gene that is appropriate to normalize gene expression in different organisms and conditions^21, 22^. To facilitate the validation of reference genes, four models based on different statistical algorithms, Genorm^20^, NormFinder^23^, BestKeeper^24^ and delta-Ct^25^ were combined in a free web tool, RefFinder^26^. RefFinder provides an overall final ranking based on the calculation of the geometric mean of each program to estimate the stability of the candidate reference genes^26^.

The current studies were designed to identify and validate the RNAi machinery of *A. glabripennis*, as well as to identify reliable reference genes for normalization in gene expression studies. *In silico* analysis was performed to identify genes involved in RNAi pathways, based on their homology to sequences of RNAi genes identified in other insects. This was then followed by validation of a reliable reference gene and the profiling of *iap* (inhibitor of apoptosis) gene expression. RNAi bioassays were performed with both ALB larvae and adults using dsRNA targeting *iap* gene. Injection of dsRNA targeting *iap* (dsIAP) into larvae and adults caused knockdown of target gene and death of both larvae and adults. This is the first study to provide evidence for RNAi response and validation of reference genes for expression analyses for ALB. These data could help future studies on gene expression as well as the development of RNAi-based methods against this and other invasive wood-boring insects.

## Results and Discussion

### Identification of ALB RNAi genes

**Figure 1:**
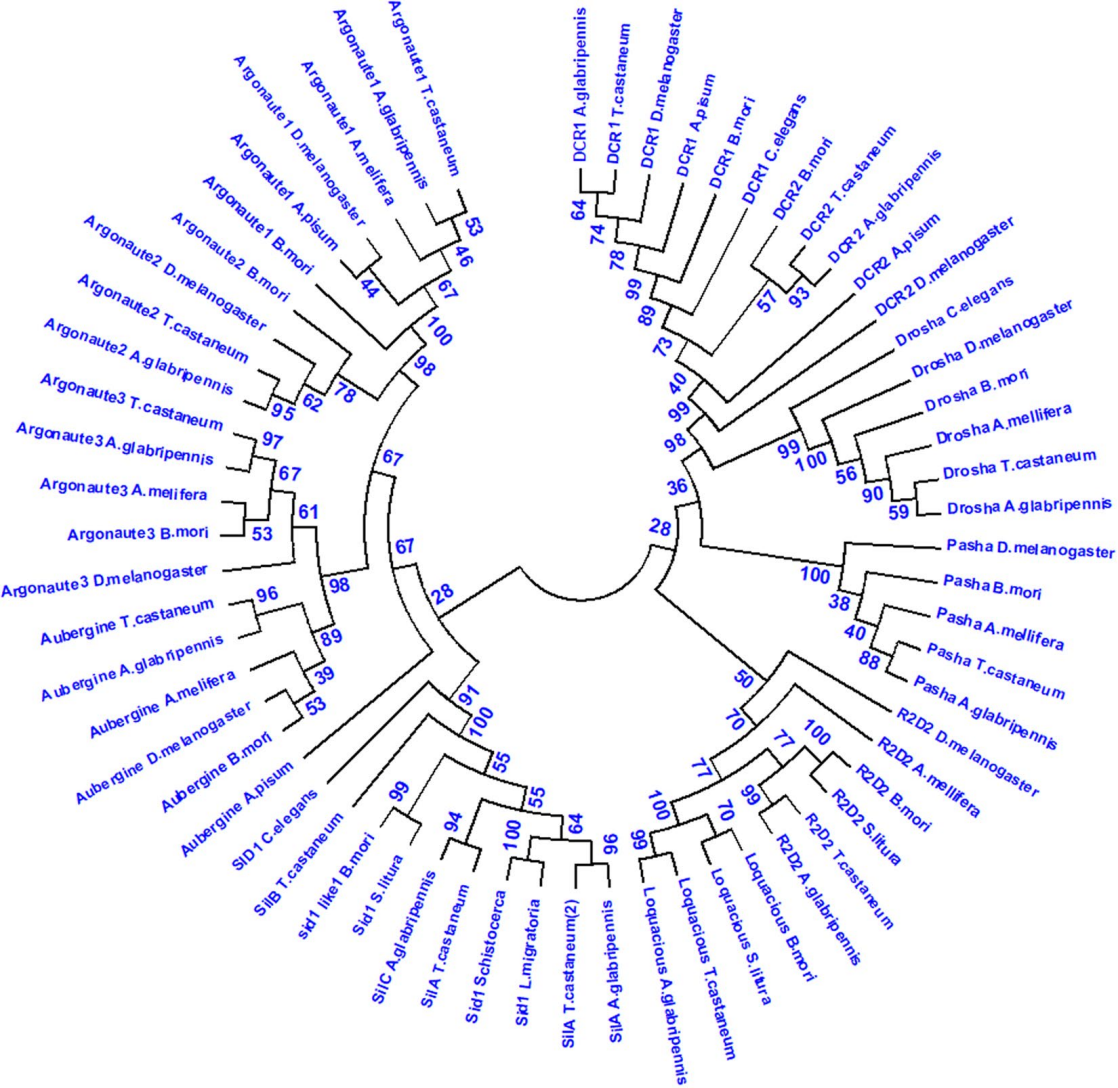
Phylogenetic tree of RNAi genes. The software MEGA 7.0 was used to construct the phylogenetic tree. Neighbor-joining method was performed and bootstrapping was used to estimate the reliability of phylogenetic reconstruction (5000 replicates).

A total of 33 RNAi genes were identified in the genome of ALB (Table 1). The genes coding for proteins involved in siRNA (Dicer 2, R2D2, Ago 2), miRNA (Dicer 1, Drosha, Loqs, Pasha, Ago 1), and piRNA (Aubergine, Ago 3) pathways were identified. The Dicer proteins contain two RNAse III, helicase, Dicer, Paz, and dsRNA binding domains. Argonaute proteins contain PAZ and PiWi domains. Drosha contains two RNase III domains. Loquacious, R2D2, and Pasha contain three, two and one dsRNA binding domains, respectively. A phylogenetic tree was constructed by comparing the sequences genes identified in ALB with their homologs in other organisms (Fig. 1).

**Table 1:** RNAi genes identified in the genome of *A. glabripennis* and their predicted biological function.

Group	Genes	ALB Accession	Accession #	Identity	Biological function	Reference
**Core RNAi machinery**	Dicer 1	AGLA015685-PA	TC001750	71%	RNase III, conversion of pre-miRNA to miRNA	9
	Dicer 2	AGLA013917-PA	TC001108	55%	RNase III, processing of long dsRNA into siRNAs	9
	Ago1	AGLA017309-PA	TC005857	99%	Catalytic subunit of RISC	9
	Ago2	AGLA012768-PA	TC011525	60%		9
	Ago3	AGLA010388-PA	TC008511	56%		38
	Piwi/Aub	AGLA006316-PA	TC008711	64%		38
	Drosha	AGLA019222-PA	TC016208	92%	RNase III, cleavage of pri-miRNA to pre-miRNA	9
	R2D2	AGLA013618-PA	TC008716	44%	dsRNA binding co-factor of Dicer-2	9
	Pasha	AGLA000778-PA	TC015332	69%	miRNA biogenesis	39
	Loquacious	AGLA003298-PA	TC01166	56%	dsRNA binding co-factor of Dicer-1	9
**Vesicle mediated transport**	Arf72A	AGLA012021-PA	TC008443	98%	Endosome transport	40
	AP50	AGLA021602-PA	TC011923	99%	Endocytosis	
	Clathrinhc	AGLA019383-PA	TC015014	97%	Endocytosis	
	Rab7	AGLA012748-PA	TC006036	76%	Endosome transport	
**Proton transport**	Vha16	AGLA014759-PA	TC011025	92%	ATP synthase/ATPase	40
	VhaSFD	AGLA018530-PA	TC006281	85%	ATP synthase/ATPase	
**Intracellular transport**	IdICP	AGLA000215-PA	TC010886	60%	Exocytosis	40
	Light	AGLA019229-PA	TC015204	85%	Lysosomal transport	
	NinaC	AGLA004194-PA	TC014087	37%	Rhodopsin mediated signaling	
**Lipid metabolism**	Gmer	AGLA010431-PA	TC006281	78%	Metabolism	40
	P13K59F	AGLA002972-PA	TC000620	79%	Lipid metabolism	
	Saposinr	AGLA006229-PA	TC000449	62%	Lipid metabolism	
**dsRNA uptake proteins**	SilA	AGLA015910-PA	TC011760	52.4%	dsRNA transporter	41
	SilC	AGLA005113-PA	TC015033	59.9%		
**Miscellaneous proteins**	Egghead	AGLA003739-PA	TC008154	84%	Oogenesis	40
	Eater	AGLA015417-PA	TC030481	38%	Innate immune response/phagocytosis	42
	Scavenger receptor	AGLA006342-PA	TC015640	53%	Endocytosis	40
	RNA helicase DDX	AGLA008916-PA	TC010546	50%	DEAD-box RNA helicase, required for RNAi	43
	Epsin-like (rsd-3)	AGLA017225-PA	TC012168	64%	Systemic RNAi	44
	Liquid facets (Epn-1)	AGLA021257-PA	TC005393	97%	Endocytic protein	11
**dsRNase enzymes**	dsRNase1	XP_018564211	XP_015840884	45%	Extra cellular endonuclease	31
	dsRNase2	XP_018561279	AEE63490.1	48%		
	dsRNase3	XP_018579804	XP_970494.1	41%

The presence of genes coding for proteins involved in the transport of dsRNA into and within the cells as well as to spread the silencing signal throughout the organism is very important for successful RNAi. Discovered in *C. elegans*, systemic RNA interference deficient (SID) proteins are essential and sufficient to mediate uptake and systemic spread of RNAi signal^27^. While SID-1 homologous genes have been identified in some insects, SID-2 and SID-5^28^, and the tyrosine kinase SID-3^29^ have not been reported in insects. In ALB, we identified two Sid1-like proteins (Sil) homologous to SilA and SilC in *Tribolium castaneum*. An additional Sil (SilB) protein was identified in *T. castaneum* and *Bombix mori* ^11^. However, we did not find SilB homolog in ALB. Whether or not SilA and SilC proteins are involved in dsRNA transport in ALB requires further investigation. We also identified homologs of *T. castaneum* genes coding for proteins known to function in vesicle-mediated transport (Arf72A, AP50, and Rab7), intracellular transport (IdICP, Light, and NinaC), as well as Rsd-3, Lqf (orthologous to Epn-1/Lqf), and scavenger receptors in ALB. Some of these proteins could act as dsRNA receptors and function in dsRNA transport and systemic RNAi mechanism^11^. Other proteins identified to function in the endocytosis pathway in *Leptinotarsa decemlineata*, such as clathrin heavy chain and vacuolar H^+^ ATPase the 16 kDa subunit, subunit c (Vha16)^30^, were also identified in this study. These data showed the presence of RNAi machinery in ALB. In addition, we identified three dsRNases homologous to dsRNase genes in other insects^31^ (Table 1). In *Lygus lineolaris* (Hemiptera) dsRNases identified in saliva and completely digest long dsRNAs resulting in no-silence effects by orally administered dsRNA^32^. Further investigation of AgladsRNases including determining the expression levels of these two genes in specific tissues of this insect may uncover the role of these enzymes in the RNAi efficiency.

### Validation of reference genes

**Figure 2:**
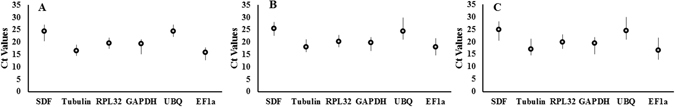
Identification of stable reference genes. Ct values of the five candidate reference genes in three independent experiments. Data obtained using RNA isolated from larval tissues (**A**) adult tissues (**B**) and pooled RNA from both larvae and adults (**C**) are shown. Black bars show the maximum (Max) and the minimum (Min) Ct values.

For normalization of gene expression in RT-qPCR, a moderately expressed reference gene is preferred because extremely high or low expression of a housekeeping gene could introduce variability in data analysis, so a standard Ct value range was analyzed for all three experiments (Fig. 2). The expression levels of all candidate genes were measured by the Ct value, which is the number of PCR cycles needed to reach a specific threshold level of detection and is inversely correlated with the quantity of initial RNA template. Succinate dehydrogenase flavoprotein subunit A (*sdf*) and Ubiquitin (*ubq*) showed lower levels of expression (Ct value 24). Tubulin (*tubulin*), ribosomal protein L32 (*rpl32*), glyceraldehyde 3-phosphate dehydrogenase (*gapdh*) and elongation factor-1 alpha (*ef1a*) showed moderate levels of expression (Ct values between 16 to 20 cycles).

Four different programs were used for analysis of reference gene expression (geNorm, NormFinder, BestKeeper, and delta-Ct method) to estimate the stability of six candidate reference genes among different tissues and development stages using a web tool that provides a reference gene stability ranking. A final ranking based on the calculation of the geometric mean of the four algorithms was generated by RefFinder, where the smaller the geometric mean, the greater the stability of reference gene expression. The first experiment compared gene expression among different larval tissues. The geNorm program was used to calculate the stability of the reference genes based on an *M* value. The lower the M value, the more stable is the expression of the reference gene, and values of *M* that surpass the cutoff value of 1.5 are not considered stable across treatments. According to this algorithm, all candidate genes had *M* ≥ 1.5. The *gapdh*, *ubq* and *ef1a* genes showed the lower *M* value of 1.03, and consequently the most stable genes. The NormFinder program analyzes both intra and inter-group variations, and lower output scores indicate the reduced variation of the reference gene expression. The *gapdh*, *ubq* and *ef1a* are the most stable reference genes with *M* value of 0.79, 0.89, and 0.97. The BestKeeper algorithm calculates standard deviation (SD), with lower values considered more stable, and values that surpass the cutoff value (SD < 1) are considered to be unstable across all treatments. This analysis indicated *rpl32* is the only gene stable among the genes tested (SD value of 0.91). The comparative delta-Ct method was used to estimate the most stably expressed reference gene based on delta-Ct value variation. A lower value is considered more stable, and the results are similar to geNorm and BestKeeper with the *gapdh*, *ubq*, and *ef1a* being the most stable with a value of 1.34, 1.39, and 1.42. The final ranking suggests that the most stable reference gene across several larval tissues is *gapdh* followed by *ubq*, *rpl32*, *ef1a*, *tubulin* and *sdf* (Table 2).

**Table 2:** Ranking of the candidate HKGs according to their stability value by geNorm, NormFinder and BestKeeper analysis.

Gene name	geNorm		NormFinder		BestKeeper		delta-CT		Comprehensive	
	M	R	SV	R	SD	R	SD	R	GM	R
**Larva Tissues**										
***gapdh***	1.037	1	0.796	1	1.045	2	1.34	1	1.19	1
***rpl32***	1.389	5	1.03	4	0.914	1	1.47	4	2.99	3
***ubq***	1.037	1	0.898	2	1.18	4	1.39	2	2	2
***sdf***	1.46	6	1.297	6	1.406	6	1.6	6	6	6
***tubulin***	1.344	4	1.211	5	1.224	5	1.54	5	4.73	5
***ef1a***	1.03	3	0.973	3	1.143	3	1.42	3	3	4
**Adult Tissues**										
***gapdh***	0.964	1	0.543	1	1.16	2	1.48	2	1.41	2
***rpl32***	0.964	1	0.549	2	1.048	1	1.46	1	1.19	1
***ubq***	1.402	4	1.372	4	1.886	5	1.84	4	4.23	4
***sdf***	1.217	3	1.3	3	1.49	4	1.8	3	3.22	3
***tubulin***	1.6	5	1.605	5	1.177	3	2	5	4.4	5
***ef1a***	1.796	6	1.866	6	2.29	6	2.19	6	6	6
**Combined larval and adult tissues**										
***gapdh***	1.24	1	0.734	1	1.089	2	1.44	1	1.19	1
***rpl32***	1.334	3	0.879	2	0.955	1	1.509	2	1.86	2
***ubq***	1.24	1	1.222	3	1.429	4	1.667	3	2.45	3
***sdf***	1.395	4	1.259	4	1.478	5	1.688	4	4.23	4
***tubulin***	1.554	5	1.351	5	1.364	3	1.76	5	4.4	5
***ef1a***	1.651	6	1.479	6	1.567	6	1.844	6	6	6

M, the gene expression stability measure; SD, standard deviation value; SV, stability value; GM, Geomean value and R, Ranking.

For different adult tissues, the geNorm statistic algorithm indicated that *gapdh* and *rpl32* are the most stable genes with an M score of 0.96, and *tubulin* and *ef1a* surpassed the cutoff value and are considered unstable, with M values of 1.6 and 1.79 respectively. The normfinder analysis also showed *gapdh* and *rpl32* are also the most two stable genes, with a value of 0.54. For BestKeeper, only *rpl32* did not surpass the cutoff value with an SD value of 1.04. The comparative delta-Ct method indicated that *rpl32* and *gapdh* are the most stable genes, with a value of 1.46 and 1.48, respectively. The geometric mean ranking picked *rpl32* and *gapdh* are the most stable genes across different adult tissues (Table 2).

Summary of data from larval, adult (male and female) tissues and stages are included in Table 3. The geNorm statistic algorithm showed that *gapdh* and *ubq* are the most stable genes with an *M* score of 1.24, and *ef1a*. The *gapdh* gene is also identified as the most stable reference gene with a stability value of 0.74 and SD value of 1.44 using NormFinder and the comparative delta-CT algorithms, respectively. The BestKeeper method indicated that *rpl32* and *gapdh* are the most stable genes, with a standard variation of 0.95 and 1.08. The final ranking calculated based on the combined algorithm values showed *gapdh*, *rpl32*, *ubq*, *sdf*, *tubulin*, and *ef1a* as the most to the least stable genes.

**Table 3:** Primers used in identification of reference genes.

Gene Name	Amplicon (bp)	Sequence 5′- 3′	R^2^	Eff%
*gapdh* - Glyceraldehyde 3-phosphate dehydrogenase	101	GTACATGCCACCACTGCAAC	0.99	100
		GTGGAGGCAGGAATGATGTT		
*ef1a* - Elongation factor-1 alpha	160	TGATGCTCCTGGACACAGAG	0.99	91
		CAGTGTGAAAGCGAGCAGAG		
*Tubulin*	187	GCCTACCACGAACAGCTCTC	0.99	98
		ACTGGATGGTACGCTTGGTC		
*rpl32* - Ribosomal Protein L32	101	GAGTCAGGAGGCGTTTCAAG	0.99	110
		GACTTTCCTGAACCCTGTGG		
*ubq*- Ubiquitin	156	TCGAAAATGTGAAAGCGAAA	0.99	93
		CACGGAGTCGAAGCACTAGA		
*sdf* - Succinate dehydrogenase flavoprotein subunit A	101	CTCTCCCGGCTTATTCTCCT	0.99	91.4
		TACATGGAGCGAATCGTCTG		

R^2^: Correlation Coefficients; Eff: Amplification efficiency.

The *gapdh* gene identified as the most stable gene across different tissues of *A. glabripennis* larvae in the current study has also been identified as one of the most stable reference genes in other insects including *Apis mellifera* ^33^, *Spodoptera exigua* and *B. mori* ^22^. Since no stable reference gene has been identified in *A. glabripennis*, the reference genes identified in these studies would help *A. glabripennis* community with the gene expression studies.

### Expression of *iap*

**Figure 3:**
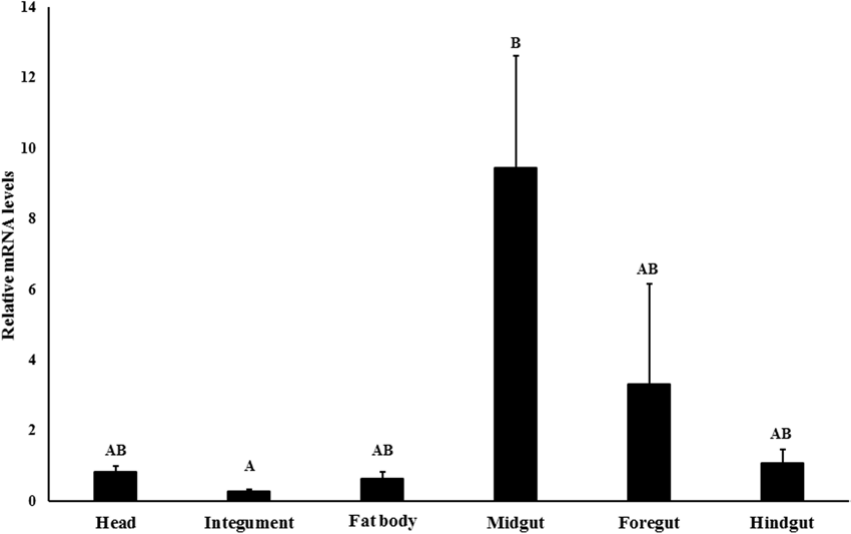
Relative IAP mRNA levels in different tissues of *A. glabripennis* larvae. Total RNA isolated from different tissues dissected from *A. glabripennis* larvae was used in RT-qPCR to quantify IAP mRNA levels using GAPDH mRNA levels for normalization. The letters show the significance of the difference in mRNA levels among tissues tested; calculated using one-way ANOVA, Tukey Test (P < 0.05, N = 3).

The expression of *iap* varied among the larval tissues tested. The highest mRNA levels were detected in the midgut followed by the foregut and hindgut. The lowest levels of IAP mRNA were detected in the integument followed by head and fat body (Fig. 3).

### RNAi-response

**Figure 4:**
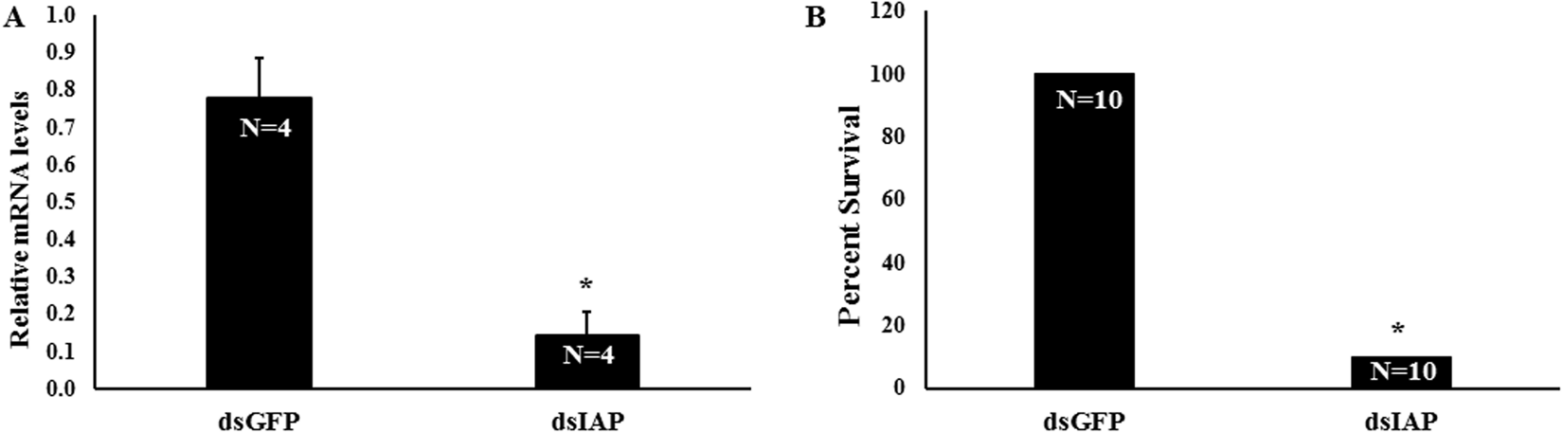
RNAi response in *A. glabripennis* larvae. (**A**) IAP mRNA levels on 5^th^ day after dsIAP injection. Total RNA isolated from *A. glabripennis* larvae injected with dsGFP or dsIAP was used in RT-qPCR to quantify IAP mRNA levels using GAPDH mRNA levels for normalization. The asterisk indicates significant differences in mRNA levels (t-test, one-tailed, P-value 0.001, N = 4). (**B**) Survival rate (%) on 10^th^ day after injection of dsRNA. The asterisk indicates significant differences in mortality between treatment and control (Fisher’s Exact test, Two-tailed, P-value < 0.001, N = 10).

**Figure 5:**
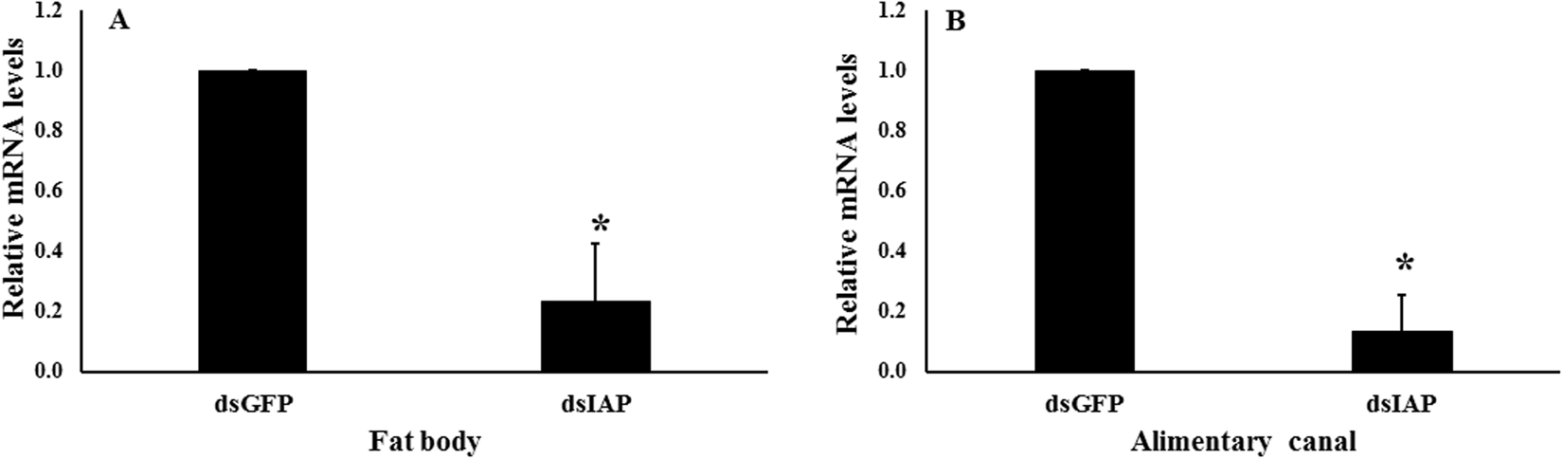
Relative IAP mRNA levels in the fat body and alimentary canal of larvae on 5^th^ day after dsIAP injection. (**A**) Relative IAP mRNA levels in the fat body. The asterisk indicates significant differences in mRNA levels (t-test, One-tailed, P-value = 0.000114, N = 3). (**B**) Relative IAP mRNA levels in the alimentary canal. The asterisk indicates significant differences in mRNA levels (t-test, One-tailed P-value = 0.00118, N = 3).

**Figure 6:**
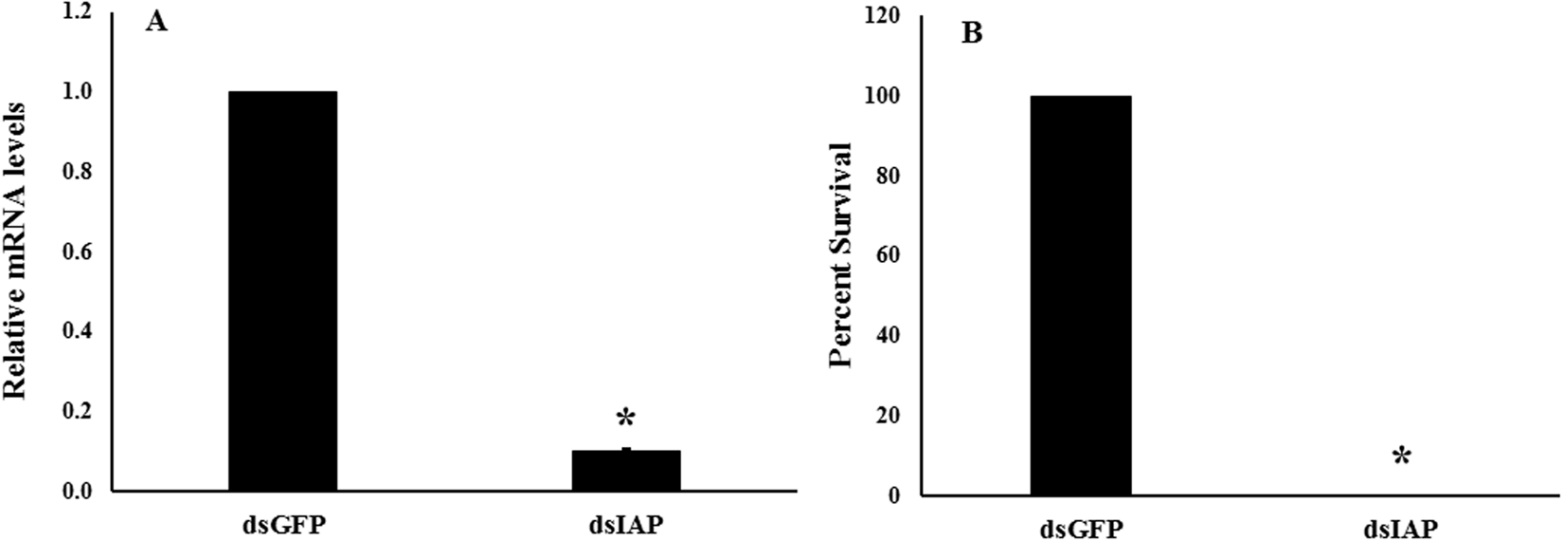
Relative IAP mRNA levels and mortality in adults treated with dsIAP. (**A**) Relative IAP mRNA levels in the adults. The asterisk indicates significant differences in mRNA levels (t-test, one-tailed P = <0.001, N = 4). (**B**) Survival rate (%) on 10^th^ day after injection of dsRNA. The asterisk indicates significant differences in mortality between treatment and control (Fisher’s Exact test, Two-tailed, P-value < 0.001, N = 10).

We prepared dsRNA targeting *iap* gene (dsIAP) and tested its efficiency in knocking down this gene in larvae. Five days after injection of dsIAP, the IAP mRNA levels were reduced by 81% when compared to their levels in control larvae injected with dsGFP (Fig. 4A). Injection of dsIAP also caused a significant mortality and only 10% of the treated larvae survived by 10 days after injection compared to 100% survival in control larvae injected with dsGFP (Fig. 4B). The knockdown of *iap* was also observed in specific tissues; fat body (77% knockdown; Fig. 5A) and alimentary canal (87% knockdown; Fig. 5B). RNAi response was also demonstrated in ALB adults. After dsIAP injection, a reduction of 91% in IAP mRNA levels was observed (Fig. 6A). The knockdown of *iap* gene in adults caused 100% mortality (Fig. 6B). Application of dsIAP caused mortality in adults and nymphs of *Lygus lineolaris* ^34^.

In conclusion, the identification of the core RNAi genes combined with RNAi bioassay results clearly demonstrates functional RNAi machinery in *A. glabripennis*. Based on our *in silico* findings, *A. glabripennis* possesses the core RNAi genes to successfully take up dsRNA and spread the signal within the insect. When dsRNA targeting an essential insect gene was injected into larvae and adults of *A. glabripennis*, nearly complete mortality of the treated insects occurred after target gene silencing in different insect tissues. These data further confirm the presence of functional RNAi machinery in *A. glabripennis*. Although the present study identified and validated the RNAi machinery in *A. glabripennis*, further studies to address the function of the AgladsRNase enzymes and their effects on RNAi response after ingestion of dsRNA need to be conducted. The characterization and understanding of the RNAi machinery could help in the development of RNAi-based methods to control this invasive wood-boring insect pest. Also, the validation of reference genes will help with the normalization in future gene expression studies.

## Material and Methods

### Identification and phylogenetic analysis of RNAi genes

Core RNAi genes coding for proteins involved in miRNA, siRNA and piRNA pathways, including vesicle mediated transport, proton transport, intracellular transport, lipid metabolism and dsRNA uptake were identified in *A. glabripennis*. The amino acid sequences of the proteins were obtained from i5K workspace (http://i5k.nal.usd.gov/webapp/blast/) based on sequence homology searches by running BLASTp using the NCBI BLAST service (http://www.ncbi.nlm.nih.gov/) and UNIPROT BLAST service (http://www.uniprot.org/). These searches used *T. castaneum* sequences as the query. Hits (contigs) obtained from these search were used to verify their sequence similarity, identity, and detection of functional domains. Multiple sequence alignment and phylogenetic tree construction were performed using the sequences identified from *T. castaneum* (Coleoptera); *Bombyx mori* and *Spodoptera litura* (Lepidoptera); *Apis mellifera* (Hymenoptera); *Droshophila melanogaster* (Diptera); *Acyrthosiphon pisum* (Homoptera); *Schistocerca americana* and *Locusta migratoria* (Orthoptera); and the nematode *Caenorhabditis elegans*. The ClustalW program in MEGA 7.0 was used to align Dicer (Dcr1, Dcr2, Drosha), dsRBP (Pasha, R2D2, Loquacious), Argonaute (Ago1, Ago 2, Ago3, Aubergine), and Sid1-like protein (SilA, SilC) sequences. The neighbor-joining analysis was performed in MEGA 7.0 with bootstrapping to estimate the reliability of phylogenetic reconstruction (5000 replicates).

### Insect rearing

*A. glabripennis* adults and larvae used in the studies were reared at the USDA-ARS Beneficial Insects Research Unit (BIIRU). The *A. glabripennis* colony was developed from beetles collected in 1999 from infested areas in New York, New Jersey, Chicago, and China and has since been maintained at 22.0 ± 2.5 °C, 45–75% RH, under a 14:10 h (L:D) photoperiod. Adult beetles were reared with fresh red maple twigs, and provided with larger logs for oviposition in 3.47-L plastic jars. To produce the appropriate instars of host larvae for tests, freshly cut red maple bolts were exposed to gravid *A. glabripennis* adults for one to two weeks in rearing jars (one female x one male per log per jar) with vented hard plastic lids under the rearing conditions mentioned above) to obtain appropriate levels of *A. glabripennis* egg deposition. Females of *A. glabripennis* chew oviposition pits and insert their eggs between the inner bark and the sap wood. The exposed logs were then incubated in the rearing room under the same environmental condition for three to four weeks until larvae hatched. Newly hatched larvae (approximately one to two weeks old after hatching) were then dissected out of infested logs and reared on artificial diet in 50 ml cup for four to six weeks before use in various experiments.

### RNA extraction, primer design, and RT-qPCR

Total RNA was isolated from dissected tissue or whole larvae using the TRI Reagent RT (Molecular Research Center Inc.). The cDNA was synthesized using M-MLV Reverse Transcriptase (Invitrogen) from RNA and was used as a template in RT-qPCR. Candidate reference genes were selected based on the previous publications reporting stability in other insects^21, 35, 36^. Gene specific primers for each gene were designed using Primer3Plus (http://www.bioinformatics.nl/cgi-bin/primer3plus/primer3plus.cgi). Sequences of primer are included in Table 4. PCR amplification efficiencies (E) and correlation coefficients (R^2^) were checked to validate the RT-qPCR primers. Standard curves were constructed using 5-fold serially diluted cDNA, a mix of female and larva heads, for each primer pair. The expression analyses of the target genes were conducted using SYBR Green PCR Master Mix. Briefly, the PCR mixture contained 1 μL of synthesized cDNA, 0.2 μL of each primer (10 mM), 5 μL of the SYBR green PCR master mix and 3.6 μL of ddH_2_O. The reactions were carried out in triplicate per template in a final volume of 10 μL. RT-qPCR reactions were performed on the StepOnePlus Real-Time PCR system (Applied Biosystems) using the following cycling conditions: one cycle at 95 °C, followed by 40 cycles of denaturation at 95 °C, annealing and extension at 60 °C. At the end of each RT-qPCR reaction, a melting curve was generated to confirm a single peak and rule out the possibility of primer-dimer and non-specific product formation. For *iap* gene expression and gene silencing analyses, the 2^−ΔΔCt^ method^37^ was used to calculate the relative expression level of the target gene in the samples as compared to reference samples. For statistical analyses, one-way ANOVA, Tukey Test (P < 0.05) was used to test significance in differences of *iap* gene expression among different tissues.

**Table 4:** Primers used in RT-qPCR and dsRNA synthesis.

Primer Name	Amplicon (bp)	Primer Sequence (5′- 3′)
IAP - dsRNA - F	386	TAATACGACTCACTATAGGGTCCTCGCCGACAAAATAATC
IAP - dsRNA - R		TAATACGACTCACTATAGGGCCTCGGAATGATCGTGTTCT
IAP - RT-qPCR - F	126	GCCCAAATCGTTAAAGCAAA
IAP - RT-qPCR - R		TCGTCATCCTCTTCCCAATC

### Selection of reference genes

A web based tool, RefFinder (http://fulxie.0fees.us/?i=1)^26^, which integrates all four software algorithms, GeNorm^20^, NormFinder^23^, BestKeeper^24^ and the comparative delta-Ct method^25^ was used to evaluate reference gene stability from the experimental datasets. The mean Ct value of each sample and for each primer was used as the input data.

### dsRNA synthesis

Template DNA for dsRNA synthesis was amplified using the gene specific primers (Table 4). PCR conditions used were 94 °C for 4 min, followed by 35 cycles of 94 °C for 30 s, 60 °C for 30 s and 72 °C for 45 s, finishing with an extension step at 72 °C for 10 min. The PCR template was purified using a PCR purification kit (Qiagen). After PCR purification, dsRNA synthesis was performed using the MEGAscript RNAi Kit (Ambion Inc.) following manufacturer’s instructions. Briefly, 200 ng of purified PCR product was used as template in a 20 μL *in vitro* transcription reaction. The reaction mix was incubated for 16 h at 37 °C, followed by 15 min of DNase treatment. The dsRNA was precipitated by adding 0.1 x volume of sodium acetate (3 M, pH 5.2) and 2.5 x volume of 100% ethanol and incubation at −20 °C for at least 2 h followed by centrifugation at 4 °C for 30 min. The dsRNA pellet was then rinsed with 750 μL of 75% ethanol and centrifuged again at 4 °C for 15 min. The ethanol was removed and the dsRNA was dried and diluted in ultrapure distilled water. The quality of the dsRNA was checked by electrophoresis and quantified using a spectrophotometer (NanoDrop Technologies). dsRNA targeting a fragment of the gene coding for green fluorescence protein was prepared using the protocol described above was used as a control.

### dsRNA bioassays

To assess gene silencing in *A. glabripennis* larvae, six to eight weeks-old larvae were injected with 2 μL containing 5 μg of dsRNA. For testing gene silencing in different tissues of *A. glabripennis* larvae (four to five weeks-old), and adults (two to three weeks after emergence) were injected with 2 to 2.5 μL containing 16 μg of dsRNA. Two treatments, dsIAP as a treatment and dsGFP as a control, were performed. After injection, adult beetles were maintained on freshly cut maple twigs and larvae were maintained on artificial diet in a plant growth chamber under normal rearing conditions described above. On the fifth day after dsRNA injection, the insects were collected and stored at −80 °C until RNA extraction. For statistical analysis, t-test [One-tailed (P ≤ 0.001)] was used for *iap* gene silencing compared to a single control (dsGFP injected). For the mortality bioassay, larvae (four to five weeks-old) and adults (one to two weeks-old) were injected with approximately 16 μg of dsRNA (in 2–2.5 μl) targeting *iap* or *gfp* gene. The mortality was scored on the 10^th^ day after the injection. For statistical analysis, Fisher’s Exact test [Two-tailed (P < 0.001)] was performed.
